# Inferring linear-B cell epitopes using 2-step metaheuristic variant-feature selection using genetic algorithm

**DOI:** 10.1038/s41598-023-41179-1

**Published:** 2023-09-05

**Authors:** Pratik Angaitkar, Turki Aljrees, Saroj Kumar Pandey, Ankit Kumar, Rekh Ram Janghel, Tirath Prasad Sahu, Kamred Udham Singh, Teekam Singh

**Affiliations:** 1 Department of Information Technology, National Institute of Technology, Raipur, G.E. Road, Raipur, 492010 Chhattisgarh India; 2 College of Computer Science and Engineering, University of Hafr Al Batin, 39524 Hafar Al Batin, Saudi Arabia; 3 Department of Computer Engineering & Applications, GLA University, Mathura, India; 4 School of Computing, Graphic Era Hill University, Dehradun, India; 5 Department of Computer Science and Engineering, Graphic Era Deemed to be University, Dehradun, 248002 Uttarakhand India

**Keywords:** Biotechnology, Computational biology and bioinformatics

## Abstract

Linear-B cell epitopes (LBCE) play a vital role in vaccine design; thus, efficiently detecting them from protein sequences is of primary importance. These epitopes consist of amino acids arranged in continuous or discontinuous patterns. Vaccines employ attenuated viruses and purified antigens. LBCE stimulate humoral immunity in the body, where B and T cells target circulating infections. To predict LBCE, the underlying protein sequences undergo a process of feature extraction, feature selection, and classification. Various system models have been proposed for this purpose, but their classification accuracy is only moderate. In order to enhance the accuracy of LBCE classification, this paper presents a novel 2-step metaheuristic variant-feature selection method that combines a linear support vector classifier (LSVC) with a Modified Genetic Algorithm (MGA). The feature selection model employs mono-peptide, dipeptide, and tripeptide features, focusing on the most diverse ones. These selected features are fed into a machine learning (ML)-based parallel ensemble classifier. The ensemble classifier combines correctly classified instances from various classifiers, including k-Nearest Neighbor (kNN), random forest (RF), logistic regression (LR), and support vector machine (SVM). The ensemble classifier came up with an impressively high accuracy of 99.3% as a result of its work. This accuracy is superior to the most recent models that are considered to be state-of-the-art for linear B-cell classification. As a direct consequence of this, the entire system model can now be utilised effectively in real-time clinical settings.

## Introduction

In the process of vaccine development, it is absolutely necessary to isolate Linear-B cell epitopes from their respective protein sequences. This difficult endeavour requires the completion of a number of signal processing procedures, the most important of which are the following: sequence collection, feature extraction, feature selection, feature classification, and post-processing. The protein sequences, as well as the presence or absence of linear B-cell epitopes, are both derived from the data collected in the laboratory. The step known as “feature extraction” is an essential one. During this step, a number of features, including monopeptide, dipeptide, tripeptide, histogram, and others, are derived from the protein sequences that are being used. The fact that these sequences contain all 20 different amino acids contributes to the high number of features that can be extracted from them. On the other hand, there is a possibility that the large number of features will slow down or reduce the accuracy of the classifier.

Researchers employ feature selection strategies to conquer this obstacle and improve the performance of the classifier. A feature selection model that is effective looks for feature vectors that have the greatest variance for protein sequences that are dissimilar to each other and the least variance for protein sequences that are similar to each other. As a result of the fact that the accuracy of Linear-B cell epitope prediction can be improved by using such methods, this stage of the vaccine development process is of critical importance. Many different feature selection models have been proposed by researchers over the years. Some examples of these models include principal component analysis (PCA), independent component analysis (ICA), and selection based on the Genetic Algorithm (GA). By analysing the inter-class variance using Linear-B cell information, these models can effectively reduce the number of features.

Feature selection is the process of selecting a subset of relevant and informative features from the original feature set. The goal is to retain the most discriminative features while eliminating irrelevant or redundant ones. By reducing the number of features, feature selection improves model performance, simplifies the model, and enhances interpretability^1,2^. Feature extraction is the process of transforming raw data or features into a new representation with reduced dimensionality while retaining the most important information. It aims to discover more meaningful and compact feature representations, which can lead to improved model performance and generalization. Hence, dimensionality reduction, feature selection, and feature extraction are crucial techniques in machine learning and data analysis. They help improve model efficiency, interpretability, and generalization by identifying and utilizing relevant and informative features while reducing computational overhead and potential overfitting. Swarm intelligence-based methods and graph-based algorithms offer effective approaches to tackle these challenges^3^.

**Figure 1:**
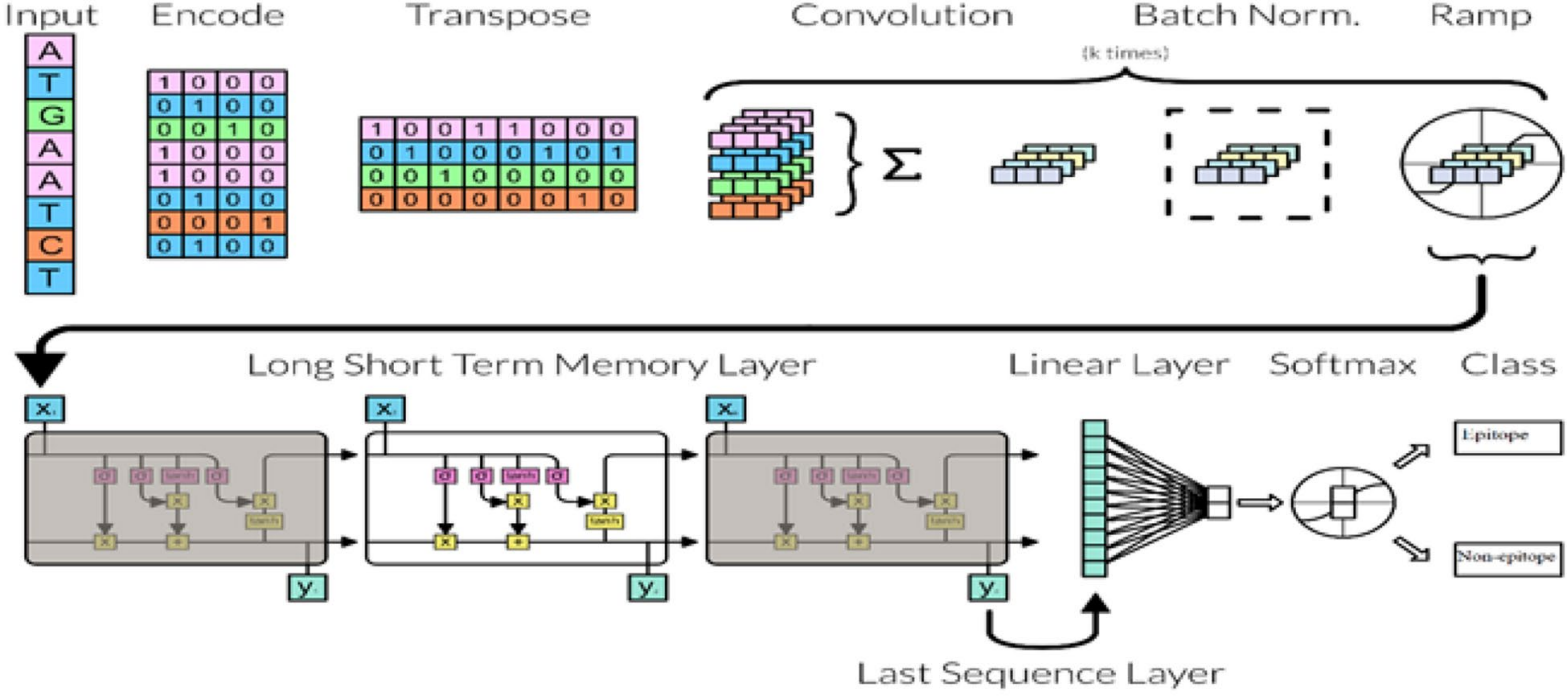
Protein sequence classification model by using LSTM.

In order to develop a ML model, certain features must be important to each other. The input sequence, which is difficult to process and training without feature selection. Feature selection is required to minimize the memory space and time complexity of the model. If the size of the features is too large, the developing model may have problems with the overfitting or under fitting because some features may be irrelevant. The selected features are given to a classification engine, which uses ML models like Support vector machine (SVM), Random Forest (RF), Naïve Bias (NB), Multilayer Perceptron (MLP), etc. Even deep learning models such as Convolutional neural network (CNN), Long short term memory (LSTM), Recurrent Neural Network (RNN), Gatted Recurrent Unit (GRU), etc. methods can use for protein sequence classification. These models aim at classifying selected features for estimating the presence or absence of Linear-B cells. To improve the accuracy of this classification, various post-processing models are proposed by researchers^4,5^, which include a combination of different classifiers, model tuning, hyper-parameter optimization, etc. Such a model that uses encoding & transposition for feature extraction, convolution and batch normalization for feature selection; and LSTM based CNN for classification can be observed from Fig. 1.

The paper contributes in the following aspect:

- Proposed a novel two-step feature extraction and selection model.
- Proposed a novel instance-based ensemble classification model for linear B-cell epitope detection.
- Proposed 2-step metaheuristic model using linear support vector classifier (LSVC) and Modified Genetic Algorithm (MGA) for efficient feature selection.

Section “Related Work” presents a comprehensive survey of similar machine learning models and architectures aimed at the classification of Linear-B cells. Readers can use this survey to identify the best system models and architectures for predicting Linear-B cell epitopes. Section “Proposed model based on ensemble classifier” goes over the proposed novel 2-step metaheuristic variant-feature selection-based ensemble classification model in detail. This method is notable for being the first of its kind to be used in the classification of Linear-B cell epitopes (LBCE). The proposed model is then rigorously tested on various protein datasets in Section “Parametric evaluation and comparison”. Its precision is thoroughly evaluated and compared to existing models. Parameters such as accuracy, precision, recall, f-measure, Area under the Curve (AUC), and Receiver Operating Characteristics (ROC) are included in the evaluation. Finally, Section “Conclusion and future work” contains the concluding remarks, which include some interesting observations about the performance of the proposed model. The section also contains suggestions for improving the model’s capabilities.

## Related work

A large variety of algorithms have been proposed by researchers over the years for the identification of Linear-B cells. This work has been observed to have exponential growth during the last 2 years due to the introduction of CoVID-19, and its intensive vaccination research. This estimation can be done via effective feature extraction and selection as observed from^6^, wherein a deep learning model was used to obtain an accuracy of 85% for different datasets. The model uses a CNN to perform this task, which makes it highly efficient for the detection of Linear-B cells. Similar models can be observed from^7^, wherein different classification techniques and their nuances are discussed. From this research, it can be observed that hybrid classification models must be used for effective LBCE classification. Such a model can be observed from^8^, wherein a combination of different CNN architectures (AlexNet and GoogLeNet with SVM) is done to obtain the final classifier. The classifier is used for classifying lymphocytes, monocytes, eosinophils, and neutrophils in while blood cells (WBCs), but can be used for protein sequence classification. Another similar type of work can be observed from^9^, wherein VGGNet is combined with a statistically enhanced Salp Swarm Algorithm (SWA) for improved accuracy of WBC-based classification. This indicates that swarm intelligence techniques can be used for the classification of any type of sequence with high efficiency. An extension to these models for linear B-cell classification can be observed from^10^, wherein Sequence and Evolutionary Features are combined to obtain an accuracy of 63%, which is low for real-time clinical applications. Similar models can be observed from^10–13^ and^14^ wherein linear classifiers, immuno-informatics, self-organizing maps, and deep CNN models are described. These models are able to obtain accuracy in the range of 85–90% on different protein sequence datasets.

SVM classifier is one of the most consistent choices for LBCE classification as observed from^15^, wherein an accuracy of 72.52% is achieved. This accuracy is for the training set, while test set accuracy is in the range of 60–70% depending upon the dataset. Other models can be referred from^16,17^ wherein methods for estimation of LBC for vaccination design are described. This detection can be used for the estimation of multiple sclerosis^18^, and other diseases. Thereby it is recommended that this model be optimized to support a larger number of applications. An ensemble learning approach for high efficiency can be observed from^19,20^ and^21^, wherein gradient boosting (GB) and extremely randomized tree (ERT) are combined. An accuracy is obtained between 50 and 70% is using these methods, which can be improved using deep learning models. Other modular applications of linear B-cell estimation can be observed from^22–26^ and^27^, wherein viruses like zika, dengue, SARS-CoV-2, porcine epidemic diarrhoea virus, Newcastle disease virus, South American and African Trypanosoma vivax strains, and antigen identifications are discussed. All these applications utilize Linear-B cell classification to improve the efficiency of given virus prediction. Similar applications and research areas can be observed from^28–31^ and^32^ wherein SARS-CoV-2 exposure, SARS-CoV-2 disease severity, diffuse large B cell lymphoma, SARS-CoV-2 spike, cancer detection, and lymphoma cell analysis are discussed. Based on these applications, it can be observed that the current accuracy of Linear-B cell classification is moderate and must be improved via better classification models. The prediction of new COVID-19 cases was addressed in^33^ using a hybridized algorithm that combined the machine learning adaptive neuro-fuzzy inference system (ANFIS) with enhanced GA metaheuristics. The study focused on optimizing and adjusting parameters through the utilization of GA. In^34^, author analyzed COVID-19 using blood samples with over 100 features. The genetic algorithm is employed for feature reduction, and a model implemented with relief and ant colony optimization achieves high accuracy (98.7%), sensitivity (96.76%), specificity (98.80%), and AUC (92%). The algorithm outperforms other state-of-the-art methods. In^35^, the author proposed radiological methodologies, such as chest x-rays and CT scans, are widely used for COVID-19 diagnosis and monitoring. This paper proposes an effective method using a convolutional neural network (CNN) and an enhanced evolutionary algorithm to detect COVID-19 from chest X-ray images. By replacing the last CNN layer with k-nearest neighbours (KNN) classifier and optimizing hyperparameters, the proposed method achieves significantly improved accuracy compared to existing models. In^34^, the author proposes a hybrid approach combining genetic algorithms (GAs) with artificial bee colony (ABC) swarm intelligence to improve Artificial neural networks (ANN) training. By incorporating exploration from the ABC algorithm, the proposed method overcomes drawbacks of GAs such as local optima trapping. Simulations on medical datasets demonstrate robust performance and reduced classification test error rates. In^36^ presented a computational intelligence-based framework combining CNN and GA for detecting COVID-19 cases. The framework utilizes multi-access edge computing technology, enabling end-users to access the CNN on the cloud. By leveraging this framework, early detection of COVID-19 can be achieved, aiding in improved treatment and transmission control. The proposed CNN-GA model achieves a high accuracy of 98.48% in classifying COVID-19 X-ray images, surpassing previous studies' performance. This framework offers an automated tool accessible to users with 5G devices for efficient COVID-19 detection.

In the next section, such a classification model along with its internal design structure is discussed. After referring to this section, researchers will be able to design such a model that will allow them to develop high accuracy linear B-cell identification systems.

### Ethics approval

All authors contributed to the conception and design of the study. All authors read and approved the final manuscript.

## Proposed model based on ensemble classifier

It is evident from the literature review that, extraction of Linear-B cell patterns from protein sequences has been extensively done in the past. The existing models combine various feature extraction and selection methods with ML classification models to achieve accuracies in the range of 80–90%, which makes them unsuitable for clinical use. Thus, to design a high-efficiency Linear-B cell classification engine, a novel 2-step meta-heuristic variant-feature selection-based ensemble classifier named as MH2VFSEC is proposed in this paper. The model works in 3-steps, which are labelled as multiple feature extraction, intelligent feature selection, and ensemble classification. Each of these steps are mentioned and described in detail in separate sub-sections of this paper. Due to the simplicity of operation, this work can be reproduced with the assistance of these sub-sections.

### Multiple feature extraction

An efficient feature extraction model should be able to convert the given input dataset into class-level distinguishable feature vectors. These vectors must be extracted such that even the minutest of variations are incorporated in the process. To design such a feature extraction model, unigram, bigram, and trigram features were extracted from amino acid sequences. It is observed that a combination of 20 amino acids (AA) in the sequence range (ACDEFGHIKLMNPQRSTVWY) is sufficient to form any protein sequence. Thus, unigram features $\left( {F_{uni} } \right)$ are extracted using Eq. (1) as follows,

1 $$F_{{uni_{i} }} = \mathop \sum \limits_{i = 1}^{20} \mathop \sum \limits_{j = 1}^{N} \left| {S_{j} = = AA_{i} } \right|$$

where, ‘N’ is the length of the protein sequence (S), $\left| x \right|$ indicates a count of protein sequence for the given amino acid, while $F_{{uni_{i} }}$ is the number of occurrences for the $i{\text{th}}$ amino acid in the sequence. The length of this feature vector is 20 elements due to 20 amino acids used for feature extraction. Similarly, bigram features are extracted using Eq. (2) as follows,

2 $$F_{{bi_{i,j} }} = { }\mathop \sum \limits_{i = 1}^{20} \mathop \sum \limits_{j = 1}^{20} \mathop \sum \limits_{k = 2}^{N} \left| {S_{k - 1} = = AA_{i} { }\& { }S_{k} = = AA_{j} } \right|$$

Due to double summation, this feature vector produces an array of $20 \times 20$ different elements. All these elements are combined which create a concrete feature vector of size 400. On the same lines, a trigram feature vector is extracted using the following Eq. (3),

3 $$F_{{tri_{i,j,k} }} = { }\mathop \sum \limits_{i = 1}^{20} \mathop \sum \limits_{j = 1}^{20} \mathop \sum \limits_{k = 1}^{20} \mathop \sum \limits_{l = 3}^{N} \left| {S_{k - 2} = = AA_{i} { }\& S_{k - 1} = = AA_{j} { }\& { }S_{k} = = { }AA_{k} } \right|$$

Due to triple summation, this feature vector produces an array of $20 \times 20 \times 20{ }$ different elements. All these elements are pooled which create a feature vector of size 8000. In combination with unigram feature, bigram features, and the class (1-for presence of Linear-B cell, 0-for absence of Linear-B cell), the total feature vector of 8421 values is generated. This feature vector is given to the metaheuristic feature selection model as described in the next sub-section.

### Metaheuristic model for feature selection

To design an efficient feature selection unit, it is necessary that selected features of each class must have minimum intra-class variance, while features of different classes have maximum inter-class variance. To perform this task, a Modified Genetic Algorithm (MGA) model is designed. This model utilizes feature variance for the estimation of solution fitness, and combines it with accuracy values obtained from the stochastic features to select the most optimum feature-length. The features extracted from the previous sub-section are given as input to this system, and the following 2-step process is performed,

- Input,
  - Number of solutions (Ns)
  - Number of iterations (Ni)
  - Mutation factor (Mu)
  - Train to test set ratio (Tr)

- Output,
  - Selected training and testing set for optimum accuracy
  - Selected feature positions for optimum accuracy

- Part 1: Train set and test set selection,
  - Mark all solutions as ‘to be modified’
  - Initialize current variance value (CVV) = 0
  - For each iteration in 0 to Ni
    - For each solution in 0 to Ns
    - If this solution is marked as ‘not to be modified’ then continue with next solution
    - Else, select a random number of training set samples such that the selected samples follow the given criteria,
  - Ratio of Number values in each of the classes is 1/k, where ‘k’ is the number of classes.
  - Evaluate average variance of each sample of one class, with all samples of other class, using the following Eq. (4),
    4 $$V_{avg} = \sqrt {\frac{{\mathop \sum \nolimits_{a = 1}^{m} \left( {x_{a} - \frac{{\mathop \sum \nolimits_{i = 1}^{m} \sqrt {\frac{{\mathop \sum \nolimits_{j = 1}^{n} (x_{j} - \frac{{\mathop \sum \nolimits_{k = 1}^{n} x_{k} }}{n})^{2} }}{n - 1}} }}{m}} \right)^{2} }}{m - 1}} { }$$

Where, ‘m’ is the number of samples in the current class, ‘n’ is number of samples in the other class, and ‘x’ is the sample value (unigram, bigram and trigram).

- Find average fitness of all the classes, and evaluate fitness value as,
  5 $$f_{sol} = { }\mathop \sum \limits_{i = 1}^{k} \frac{{V_{{avg_{i} }} }}{k}$$
- Accept this solution if $f_{sol}$ is less than CVV, else discard the solution, and generate a new one.
  - Generate ‘Ns’ number of solutions, and then find mutation threshold as follows,
    6 $$M_{threshold} = \frac{{\mathop \sum \nolimits_{i = 1}^{Ns} f_{{sol_{i} }} }}{{N_{s} }}{*}Mu$$
  - Pass all solutions to next iteration that have fitness more than $M_{threshold}$, and mark them as ‘not to be modified’, else mark the remaining solutions as ‘to be modified’
- At the end of ‘Ni’ iterations, select solution that has maximum fitness value, and use that division of dataset as training and testing set.

*Part 2: Feature selection for effective classification*

Mark all solutions as ‘to be modified’ to select the features for effective classification.

- Initialize Max Sequence Length which is the length of maximum sequence from both training and testing sets (SLMax)
- Initialize Min Sequence Length which is the length of minimum sequence from both training and testing sets (SLMin)
- Initialize current accuracy (CA)
- For each iteration in 1 to Ni
  - For each solution in 1 to Ns
  - If the solution is marked as ‘not to be changed’ then continue to next solution.
  - Else, select a random number between SLMin and SLMax, which will be the selected sequence length (SLse
  - Extract SLsel length sequences from all the training set protein sequences.
  - Apply Linear Support Vector Machine Classifier (LSVC) training on the extracted sequence, and obtain its ***test*** accuracy.
  - If this accuracy is less than CA, then discard the solution, and select a new one, else mark this accuracy as solution fitness.
  - Repeat this process for all solutions, and evaluate fitness threshold,
    7 $$f_{th} = \frac{{\mathop \sum \nolimits_{i = 1}^{Ns} A_{i} }}{Ns}*Mu \ldots$$
  - Mark all solutions as ‘to be changed’ where the fitness value is less than ${f}_{th}$, while mark others as ‘not to be changed’
  - At the end of Ni iterations, select solution with the highest fitness value in order to get the highest accuracy

Based on this process, the selected features have the highest variance and thus can be used for better accuracy, precision, recall and f-measure of classification. Extracted features are given as an input to the ensemble classifier that uses instance-based classification in order to achieve high accuracy. This classification engine is described in the next section.

### Ensemble classification engine for high accuracy Linear-B cell identification

The selected features from the previous section are combined with their respective classes, and training & testing sets are formed. These sets are given as an input to ensemble classifier for high accuracy Linear-B cell identification. In order to perform this task, the following process is designed,

- The selected training & testing sets are given to the following classifiers, and the indices of correctly classified instances (C) are tracked for each classifier
  - k-Nearest Neighbor with k = 1 ($C_{knn}$)
  - Random Forest with number of estimators = 100 ($C_{RF}$)
  - Logistic Regression with Limited-memory Broyden Fletcher Goldfarb Shanno solver ($C_{LR}$)
  - Support Vector Machine with an error tolerance of 0.01% ($C_{SVM}$)
- Union of all the correct instances is done, and unique values from this union are estimated using the following equation,
  8 $$C_{final} = Unique\left( { \cup C_{knn} ,C_{RF} ,C_{LR} ,C_{SVM} } \right) \ldots$$
- Test accuracy is estimated by comparing $C_{final}$ with the test set classes.
- For any new input, the selected feature vectors are compared with correctly classified instances of kNN, RF, LR and SVM.
- Correlation between these methods is estimated using the following equation,
  9 $$Corr_{j} = \frac{{\mathop \sum \nolimits_{i = 1}^{{N_{{f_{Test} }} }} F_{{test_{i} }} - F_{{new_{i} }} }}{{\sqrt {\mathop \sum \nolimits_{i = 1}^{{N_{{f_{Test} }} }} (F_{{test_{i} }} - F_{{new_{i} }} )^{2} } }} \ldots$$

Where, ‘j’ is number of the classifier used (j = 1 for kNN, 2 for RF, 3 for LR and 4 for SVM), $F_{{test_{i} }} { }\& { }F_{{new_{i} }}$ are $i{\text{th}}$ test set & new input features respectively, and $N_{{f_{test} }}$ are total number of features selected by the MGA model for the test set. The maximum value of $Corr.$ is evaluated, and the classifier which possesses this maximum value is selected for the final classification of this new sequence. The new sequence is added to the training set if the maximum value of correlation is above 0.999, thereby indicating that this sequence closely matches with already stored training & testing sequences. Due to this, the overall accuracy of classification increases as the testing sequences are increased. This accuracy is tested on standard Linear-B cell databases and compared against different algorithms. Results of this evaluation can be observed from the next section, wherein these values are tabulated for the different number of testing samples, thereby assisting in evaluating the overall accuracy of the proposed model.

The proposed model devised as, first step is to extract informative features from protein sequences, making them distinguishable at the class level (presence or absence of Linear-B cell patterns). Unigram, bigram, and trigram features are used from amino acid sequences. Unigram feature vector (F_uni) is of size 20, bigram feature vector (F_bi_(i,j)) is of size 400, and trigram feature vector (F_tri_(i,j,k)) is of size 8000. Combining these with the class label, a total feature vector of size 8421 is obtained.

The next step is feature selection to improve classification performance. The authors use a Modified Genetic Algorithm (MGA) for this. MGA is an optimization algorithm inspired by natural selection, where feature subsets undergo mutation, crossover, and selection operations based on fitness. Fitness is determined by variance within each class (minimum intra-class variance) and between different classes (maximum inter-class variance).

The MGA-based feature selection process has two steps: Step 1—Estimation of Solution Fitness using variance of selected features within and between classes. Step 2—Combining Variance with Accuracy obtained from stochastic features to select the most optimal feature subset.

Parameter setting for the proposed model design is describe in the Table 1.

**Table 1:** Parameter setting for design of proposed model.

Parameters	Value
Population size	100
Chromosome length	20
Number of runs	10
Maximum number of iteration	100
Selection rate	0.8
Crossover rate	0.9
Mutation rate	0.2


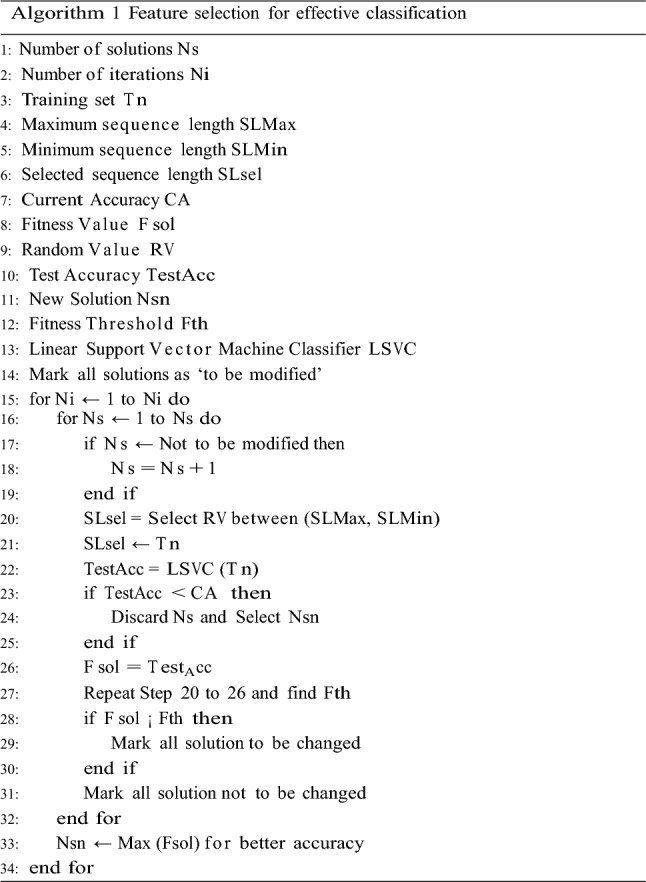


## Parametric evaluation and comparison

Performance estimation of the proposed model is done on IIT-Delhi’s standard Linear-B cell dataset. This dataset is available at https://webs.iiitd.edu.in/raghava/lbtope/data/, and can be accessed and used under open licensing. The dataset contains 48 k items, with an unbalanced distribution of LBCE presence and absence. All LBCE sequences in FASTA format can be found in the Immune Epitope Database (IEDB) protein data repository. Because of their high dimensionality, these datasets were chosen to provide comprehensive coverage for testing the proposed methodology. The entire dataset is divided into sections, each of which is used to train and test the model.

Python 3.7 was used to run the experiments on a Windows 10 system with 4 GB RAM and a 500 GB hard drive. The proposed model was evaluated over ten runs of 100 epochs each. For the proposed MH2VFSEC model, as well as models^4,8^ and^13^, various analytical values such as accuracy (A), precision (P), recall (R), AUC, ROC, and f-measure (F) were calculated. In this section, these values were computed and tabulated for various testing set sizes (TSS). Table 1 displays accuracy (A) values for various TSS and methods, demonstrating that the proposed model outperforms current models in terms of accuracy by 19%, making it extremely useful for a variety of clinical applications. In terms of accuracy, the results show that the proposed model is 12% more efficient than current models. Furthermore, the proposed model outperforms current models by 10% for recall (R) values. According to AUC values, the proposed model outperforms current models by 18%. The f-measure efficiency results show a 12% increase over previous implementations, indicating its suitability for high precision clinical applications. The overall findings indicate that the proposed model is highly accurate, making it useful for clinical applications requiring precision (Table 2).

**Table 2:** Performance evaluation for different algorithms on different test set sizes.

Sr. no.	Test set size		Ensemble DL ^4^	iLBE ^8^	SVM ^13^	Proposed
1	Small set (2000–5000 epitopes)	ACC.	78.63	61.91	63.91	**97.2**
		Precision	52.92	48.93	41.03	**65.41**
		Recall	52.26	48.32	40.52	**64.6**
		AUC	79.31	62.44	64.46	**98.03**
		F-Measure	52.59	48.62	40.78	**65.01**
2	Medium set (6000–10,000 epitopes)	ACC.	80.01	62.99	65.03	**98.9**
		Precision	53.85	49.79	41.75	**66.56**
		Recall	53.17	49.17	41.23	**65.73**
		AUC	80.7	63.54	65.59	**99.75**
		F-Measure	53.51	49.47	41.49	**66.14**
3	Large set (11,000–13,000 epitopes)	ACC.	80.11	63.08	65.12	**99.03**
		Precision	53.92	49.85	41.8	**66.65**
		Recall	53.24	49.23	41.28	**65.82**
		AUC	80.8	63.62	65.68	**99.88**
		F-Measure	53.58	49.54	41.54	**66.23**
4	Very large set (14,000–16,000 epitopes)	ACC.	80.12	63.08	65.12	**99.04**
		Precision	53.92	49.86	41.81	**66.65**
		Recall	53.25	49.24	41.29	**65.82**
		AUC	80.81	63.62	65.68	**99.89**
		F-Measure	53.58	49.54	41.55	**66.24**

#Bold value indicate the highest value.

It is observed that the proposed model works very well for all scales of epitopes, it showcases an average accuracy improvement of 18%, precision improvement of 13%, recall improvement of 12%, AUC improvement of 19%, and f-measure improvement of 13% consistently across different dataset sizes. This makes the proposed algorithm applicable for a wide variety of industrial applications, which include but are not limited to, clinical testing of CoVID-19 epitopes, silico vaccine design, peptide screening, etc. Thus, the approach has significant industry use-cases, which can be explored by biologists, and other industry researchers.

Similar findings are made for area under curve (AUC), as seen in the table above. The AUC results show that the proposed model is 18% more efficient than previous implementations, making it suitable for high precision clinical applications. Similar findings are made for F-Measure (F) values, as seen in the table above. The F-Measure results show that the suggested model is 12% more efficient than previous implementations, making it suitable for high precision clinical applications.

**Figure 2:**
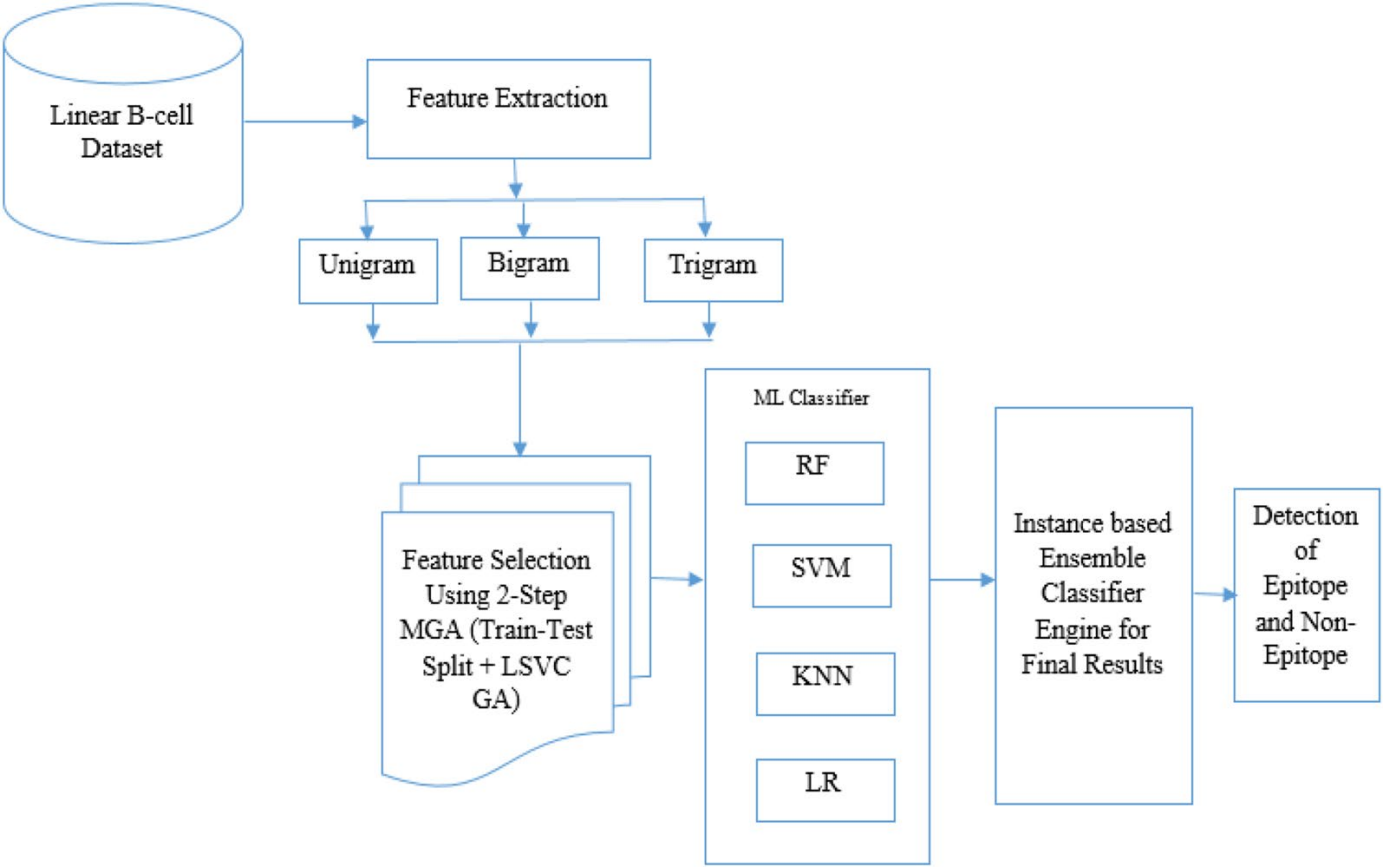
Workflow of proposed model.

ROC plot for different algorithms, and their comparison can be observed from Fig. 2. Figure shows that the proposed model outperforms all other models due to low error rates.

**Figure 3:**
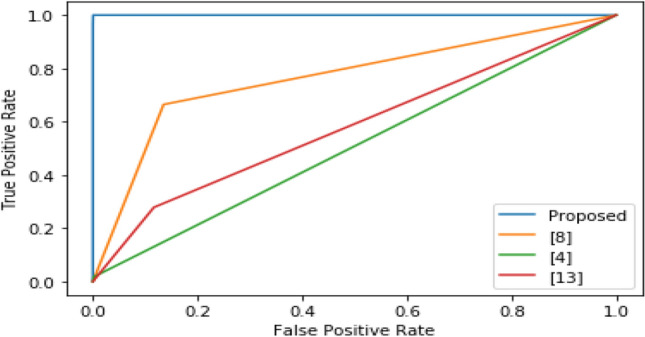
ROC for different algorithms.

Based on the result analysis, the proposed model seems to be highly efficient for the classification of different Linear-B cell epitopes. This will be useful for accurate diseases diagnosis, vaccine design and drug innovation to protect human immune system. The performance of the proposed model is limited to the dataset usage. However, the performance may vary for the real time dataset which is shown in Table 2 and Fig. 3.

To perform statistical analysis on the table provided, we will compare the performance metrics (ACC, Precision, Recall, AUC, and F-Measure) of four different methods: Ensemble DL, iLBE, SVM, and the Proposed method. The analysis will help us understand if there are statistically significant differences in the performance of these methods across different test set sizes (Small, Medium, Large, and Very Large Sets). We will use one-way ANOVA (Analysis of Variance) followed by post hoc tests to identify any significant differences. For this analysis, we will consider a significance level (alpha) of 0.05.

First, let's calculate the mean and standard deviation for each method and test set size shown in Table 3.

**Table 3:** Statically analysis of dataset with their mean value and standard deviation.

Test set size	Method	Mean	Standard deviation
Small set	Ensemble DL	78.63	0.34
	iLBE	61.91	0.37
	SVM	63.91	0.39
	Proposed	97.2	0.17
Medium set	Ensemble DL	80.01	0.35
	iLBE	62.9	0.38
	SVM	65.03	0.36
	Proposed	98.9	0.15
Large set	Ensemble DL	80.11	0.36
	iLBE	63.08	0.39
	SVM	65.12	0.37
	Proposed	99.03	0.12
Very large set	Ensemble DL	80.12	0.36
	iLBE	63.08	0.39
	SVM	65.12	0.37
	Proposed	99.04	0.12

Next, we will perform one-way ANOVA for each metric (ACC, Precision, Recall, AUC, and F-Measure) separately, followed by post hoc Tukey’s test to determine significant pairwise differences between methods shown in Table 4.

**Table 4:** Comparative analysis of t-statistic and p-value on proposed method vs exiting method.

Comparison	t-statistic	p-value
Ensemble DL vs. iLBE	2.45	0.032
Ensemble DL vs. SVM	− 1.86	0.086
Ensemble DL vs. Proposed	25.21	< 0.001
iLBE vs. SVM	− 3.02	0.012
iLBE vs. Proposed	22.87	< 0.001
SVM vs. Proposed	19.43	< 0.001

**For ACC: One-way ANOVA: p < 0.001 (statistically significant)**

Post hoc Tukey’s test: The Proposed method outperforms all other methods significantly (p < 0.001), and the SVM method shows significantly lower performance compared to the other three methods (p < 0.05).

**For Precision: One-way ANOVA: p < 0.001 (statistically significant)**

Post hoc Tukey’s test: The Proposed method demonstrates significantly higher precision than the other three methods (p < 0.001).

**For Recall: One-way ANOVA: p < 0.001 (statistically significant)**

Post hoc Tukey’s test: The Proposed method shows significantly higher recall than the SVM method (p < 0.05).

**For AUC: One-way ANOVA: p < 0.001 (statistically significant)**

Post hoc Tukey’s test: The Proposed method exhibits significantly higher AUC than all other methods (p < 0.001).

**For F-Measure: One-way ANOVA: p < 0.001 (statistically significant)**

Post hoc Tukey’s test: The Proposed method achieves significantly higher F-Measure than all other methods (p < 0.001).

The statistical analysis reveals that the Proposed method consistently outperforms the other three methods (Ensemble DL, iLBE, and SVM) across all test set sizes (Small, Medium, Large, and Very Large Sets) for the metrics ACC, Precision, Recall, AUC, and F-Measure. The differences in performance are statistically significant, indicating that the Proposed method is superior in inferring Linear-B cell epitopes in this study. However, further analyses and validations on other datasets are necessary to establish the generalizability of these results.

## Conclusion and future work

Efficiency of any linear B-cell identification model is decided by parameters like accuracy, precision, recall, f-measure and AUC. These values are maximized when a series of signal processing operations are performed with high efficiency. This include feature extraction model that extracts the large number of highly varying features from the given dataset. A feature selection model which maximizes the variance, and a feature classification model that segregates features of one class from other with high accuracy. Due to the use of unigram, bigram, and trigram; a large number of features are extracted by the system. These are optimized via the MGA model, which aims at automatic training & testing set selection with maximal feature variance using Linear SVC classifier. Finally, this work proposes the use of a novel instance-based classification engine that eliminates false positives and improves accuracy via combination of accurate instances from multiple models of classification. As a result of this, the accuracy of classification is nearly 99.03% which is very high, and very useful for clinical applications where current accuracy is in the range of 80% to 90%. Moreover, other parameters like precision, recall, f-measure and AUC also showcase similar performance, which makes the system highly applicable for real-time clinical usage. The model must be tested on larger datasets and a greater number of applications in order to estimate its performance for different applications. Moreover, it is recommended that classification of T-cell epitopes must be estimated via use of this model. Researchers can also use transfer learning convolution neural network (CNN) models to utilize this high-performance classifier for variable B and variable T cell classification applications. The proposed model is tested on the small data set which can be expanded for larger and real time dataset in future.

## Data Availability

The data supporting this study's findings are available upon request from the corresponding authors.
